# Development and Comprehensive SPE-UHPLC-MS/MS Analysis Optimization, Comparison, and Evaluation of 2,4-Epibrassinolide in Different Plant Tissues

**DOI:** 10.3390/molecules27030831

**Published:** 2022-01-27

**Authors:** Xin Liu, Yuan Zhong, Wenli Li, Guichen Li, Ning Jin, Xiaoqiang Zhao, Dan Zhang

**Affiliations:** Gansu Provincial Key Laboratory of Aridland Crop Science, Gansu Agricultural University, Lanzhou 730070, China; liux9688@163.com (X.L.); liwl@gsau.edu.cn (W.L.); lguchen@163.com (G.L.); Jinn0513@163.com (N.J.)

**Keywords:** 24-epibrassinolide, ultra-high-performance liquid chromatography–tandem mass spectrometry, maize, *Brassica napus*, marigold

## Abstract

A determination method for trace 24-epibrassinolide (EBL) in plant tissues was developed using ultra-high-performance liquid chromatography–tandem mass spectrometry (UPLC-MS/MS). The plant tissue samples were extracted using a methanol–formic acid solution, and the corresponding supernatant was purified with ODS C18 solid-phase extraction column. The extracts were separated using a Zorbax Eclipse Plus C18 (2.1 mm × 50 mm, 1.8 μm) column with methanol and 0.1% formic acid as the mobile phase. The ion source for the mass spectrometry was an electrospray ionization source with positive ion mode detection. The linear range of the target compound was 0.7~104 μg/kg, the limit of detection (LOD) was 0.11~0.37 μg/kg, the limit of quantification (LOQ) was 0.36~1.22 μg/kg, the recovery rate was 84.0~116.3%, and the relative standard deviation (RSD%) was 0.8~10.5. The samples of maize plumule, brassica rapeseed flower, and marigold leaf were detected using the external standard method. The optimization of the extraction method and detection method of EBL improved the detection sensitivity, laid a foundation for the artificial synthesis of EBL, improved the extraction rate of EBL, and provided a theoretical basis for the study of EBL in many plants.

## 1. Introduction

Brassinolide (BR) [1], a kind of steroid compound which is found widely in many plants [2], was first isolated from oilseed rape pollen extracts [3]. It plays an important role in the process of the regulation of seed germination [4], cell elongation growth [5], the plant stress response [6], and the immune responses [7], etc. BR was listed as the sixth largest new plant hormone in the 16th international conference on regulating substances (IFGSA) [8]. The brassinosteroid 24-epibrassinolide (EBL) [9,10], with the bioactive property of salt tolerance, is a by-product of the biosynthesis of BR [11]. It exhibits highly important regulatory effects during the plant life stage, especially with regard to the plant physiology. EBL has no toxic side effects on mammals, and potential medical applications have been gradually found, such as anti-cholesterol, anti-inflammation, and anti-cancer uses, etc. [12]. EBL has been widely used to promote crop growth and improve crop resistance to adversity, especially given its lower concentration in plants, high efficiency, and environmental protection properties. In recent years, there have been lots of discoveries relating to the anabolism of BR [13], its physiological function and applications [14], and the signal transduction regulatory network [15]. However, the content of EBL is lower than other plant hormones (generally at the concentration level of ng/g to pg/g), which makes the accurate qualitative and quantitative determination of EBL content difficult, and which greatly restricts the research on this plant hormone [16,17]. Additionally, the quantitative analysis of BR content in plants is of great significance when studying the mechanisms of BR.

High-performance liquid chromatography (HPLC) has been used to quantify brassinolide, which may lead to co-elution due to the complex sample matrix [18], resulting in the false positive detection of BR. The quantitative process of biological identification is complex. The enzyme-linked immunosorbent assay (ELISA) is quick, simple, and widely applicable, but the cost of the antibodies is high [19]. Pretreatment with gas chromatography (GC) and gas chromatography–mass spectrometry (GC-MS) is simple, but the derivatives are selective to the detector, so applying it widely is difficult. Additionally, ultra-high-performance liquid chromatography–tandem mass spectrometry (UHPLC-MS/MS) is highly sensitive, fast, and accurate [20]. Additionally, it can avoid the complicated derivatization process involved in other forms of analysis [21]. Using multiple reaction monitoring as a secondary screening process to obtain quantitative and qualitative data can help to accurately quantify plant trace compounds in a complex matrix. With MassHunter quantitative software (Aigilent Technologies, California, USA), accurate and quick calculations of the target compound content in a plant sample can be obtained [22]. In this paper, we provide a rapid, effective, and accurate method for the quantitative determination of EBL in plants, and provide a theoretical basis for the subsequent development of trace compound detection. By using marigold, corn, and oilseed rape as materials, an efficient solid-phase extraction column was selected through sample processing optimization, and a set of universal EBL extraction steps was optimized, which could be used for the analysis of various plant samples, providing a theoretical basis for the simultaneous detection of EBL in various plants.

## 2. Result and Discussion

### 2.1. Comparison of Sample Grinding Method

**Figure 1:**
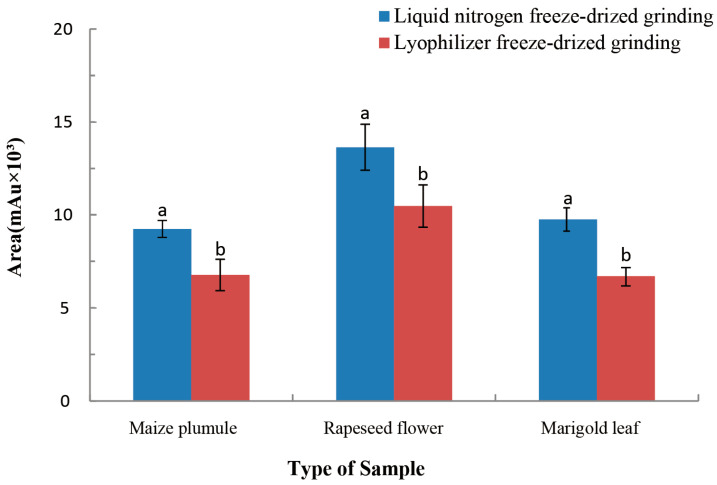
Effects of each sample crushing method on EBR detection in three plant tissues. The mean ± SEM (*n* = 3) represented by different lowercase letters showed significant differences at the 5% level using Duncan’s multi-range test. SPSS 23.0 software (IBM Corp., Armonk, NY, USA) was used for the one-way ANOVA. The significant difference levels were set at *p* < 0.05.

Firstly, 0.5 g of accurately weighed samples were processed with liquid nitrogen freeze-dried grinding and lyophilizer freeze-dried grinding. Liquid nitrogen grinding is where plant tissues are frozen in liquid nitrogen, then lyophilized, followed by being ground up (homogenizing) in a mortar and pestle. Lyophilizer grinding is a process in which plant tissues are frozen in a freezer and lyophilized in a lyophilizer and then are ground into a powder in a mortar and pestle. Each processed tissue was separated into three samples. Peak areas were compared after the sample preparation. Figure 1 shows that the peak areas of the three samples after liquid nitrogen grinding were larger than those treated by lyophilizer freeze-dried grinding. Therefore, the selection of the sample grinding method also affects the detection effect of EBL. Liquid nitrogen has a fast freezing speed and a good quick-freezing effect. Liquid nitrogen grinding results in small crystallization, a fast-drying process, and less residual water [23]. The lyophilizer freeze-dried grinding of the sample is not ideal, because the sample crystallization is relatively large in the lyophilizer, resulting in a large gap in the sample during the lyophilizer, which means that water easily escapes. When the water sublimation outside the crystal is completed, the water inside the crystal cannot easily escape, resulting in a long, time-consuming and inadequate extraction effect. Therefore, the liquid nitrogen grinding treatment is better than lyophilizer freeze-dried grinding.

### 2.2. Optimization of SPE Purification

**Figure 2:**
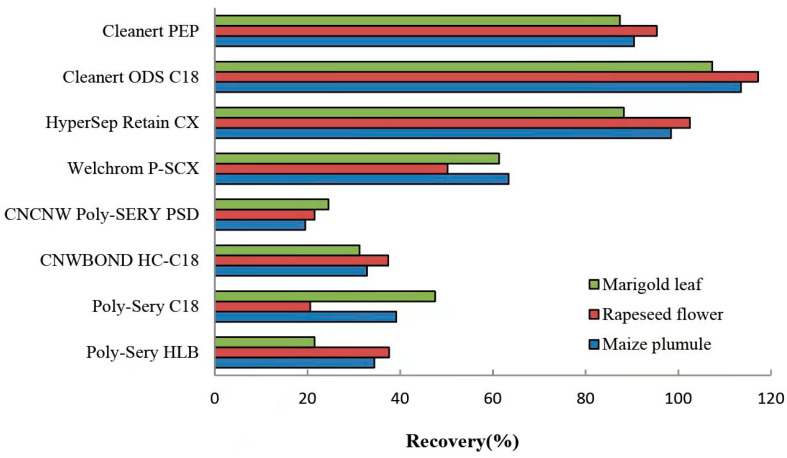
Effects of different SPE columns on EBL extraction in maize plumule.

Solid-phase extraction columns can effectively remove impurities and reduce the matrix effect of target compounds [24]. Eight commercially available solid-phase extraction columns were used to compare the enrichment and purification effects of each sample on different solid-phase extraction columns. The accurately weighed 0.5 g liquid nitrogen grounding samples were separated into control samples (without standard) and standard samples (100 μg/mL). The sample pretreatment was carried out according to the sample preparation method. The recovery rate of each sample was calculated according to the solid-phase extraction column method during sample pretreatment. The results showed that the Clearnert PEP, Clearnert ODS C18, and HyperSep Retain CX samples recovered well (Figure 2). The Cleanert ODS C18 solid phase extraction column with the highest recovery rate was selected as the final condition. The results showed that the extraction efficiency of the C18 sorbent was much higher than the others, indicating that C18 was suitable for extracting polar analytes, such as EBL.

### 2.3. The Effect of Mobile Phase

**Figure 3:**
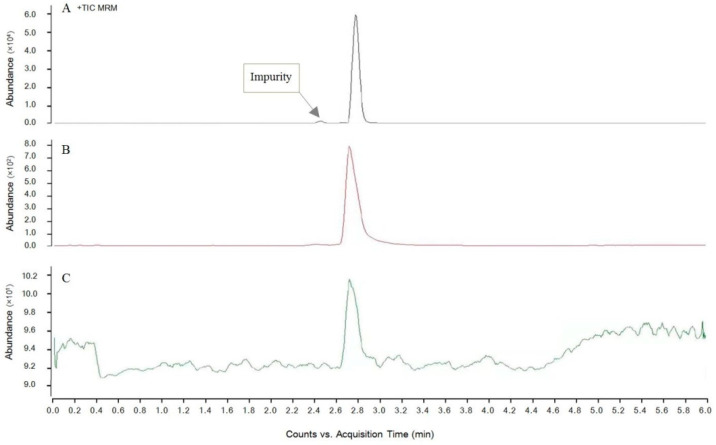
Effects of different flow rates relative to EBL standard detection. (**A**) methanol–formic acid aqueous solution, (**B**) methanol–ammonium formate aqueous solution, (**C**) acetonitrile–formic acid aqueous solution.

Three commonly used mobile phases were selected: a methanol–formic acid aqueous solution [25], a methanol–ammonium formic acid aqueous solution [26], and an acetonitrile–formic acid aqueous solution [27]. The concentration of additives was 0.1% (see Section 3.5 for an explanation of the testing conditions) and the standard substance with the same concentration was used to optimize the liquid chromatography conditions via gradient elution. As shown in Figure 3, when the methanol–formic acid aqueous solution mobile phase elution is used, the peak time detected for EBL is delayed, which facilitates the separation of the impurity peak. The peak shape is symmetrical, and the detection response value is high, while the detection limit of EBL is low and the detection sensitivity is improved. However, when using formic acid, the ammonium formic acid aqueous solution, and the acetonitrile formic acid aqueous solution, the chromatographic peak tailed, the peak time was 2.731 min, the peak type was not symmetrical, and the detection sensitivity was significantly reduced. Therefore, it is best to select a methanol–formic acid aqueous solution as the mobile phase for EBL detection of the peak type, which can help to achieve accurate qualitative and quantitative EBL, while the dwell time is 2.814 min. The total detection time of a single sample was 6.0 min. Therefore, the duty cycle is 6.0 min.

### 2.4. The Effect of Mass Spectrum Parameters

**Figure 4:**
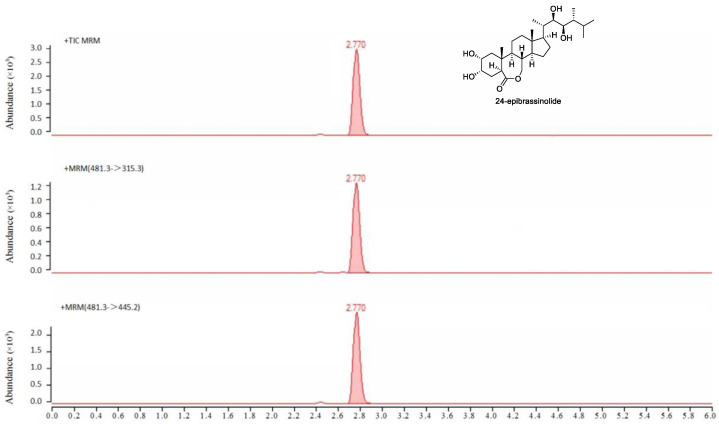
The MRM chromatogram for 24-epibrassinolide detection.

**Figure 5:**
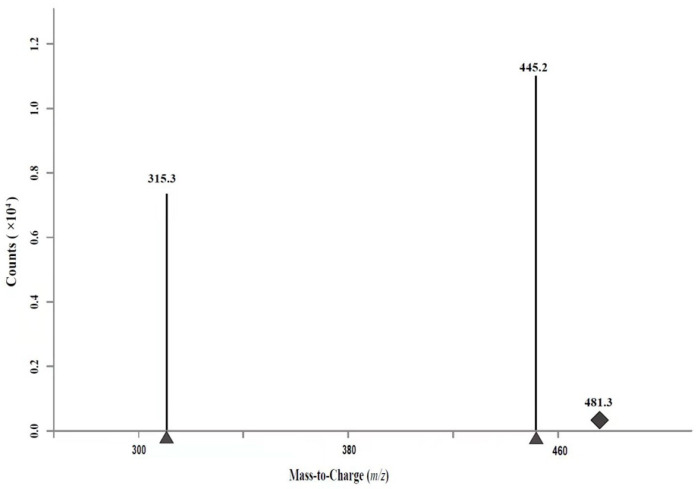
The MRM spectrometry for EBL detection.

According to the molecular formula and molecular weight of EBL, as well as the chemical and physical properties of EBL, the parent ion scanning of EBL with a standard solution concentration of 100 μg/L was carried out by using ultra-high-performance liquid chromatography–tandem mass spectrometry to optimize the detection parameters of mass spectrometry. According to the contrast of signal abundance in positive and negative ion mode, positive ion detection mode [M + H]^+^ was adopted. The parent ion of EBL was *m/z* 481.3, and the product ion was *m/z* 445.1 and *m/z* 315.1, while the collision energy and fragment were further optimized. Considering the sensitivity and selectivity of the method, a mass spectral *m/z* 445.2 transition was used to quantify EBL and a mass spectral of *m/z* 315.3 was used for confirmation. (Figure 4 and Figure 5).

### 2.5. Recovery Rate, Linearity, and Matrix Effect

The standard recoveries for EBL in corn bud, rape flower, and marigold leaf samples were determined in sample pretreatment, and the scalar values of EBL were 10, 50, and 200 μg/kg, respectively. The recoveries were 84.0–96.5%, 88.2–116.3%, and 85.6–103.4%, with relative standard deviations (RSD) of 0.9–3.4%, 3.3–10.5%, and 0.8–4.6%, respectively. This method has high accuracy and precision, and can meet the requirements of quantitative analysis for EBL in the samples.

Using the standard solution configuration method outlined in Section 3.3, UHPLC-MS/MS was used for detection. With the concentration of EBL as the X-axis and the corresponding peak area as the Y-axis, the standard curve was drawn, and linear regression was performed to obtain the regression equation and correlation coefficient (R^2^). The limit of detection for the compounds was determined with a signal-to-noise ratio (S/N) of 3 [28]. As shown in Table 1, EBL had a good linearity in the range of 0.7~104 μg/kg, with correlation coefficients of 0.9978, 0.9997, and 0.9996, and an LOD of 1.22, 0.36, and 0.95~0.37 μg/kg, respectively.

The matrix effect (ME) refers to the fact that the co-elution substances in chromatographic separation affect the ionization efficiency of the target component [3], which leads to the inhibition or improvement of the mass spectrum signal [29]. Due to the complex composition of corn bud, rape flower, and marigold leaf matrix, there may be many factors affecting EBL signal detection. The formula for ME is:

ME (%) = EBL peak area after extraction/standard EBL peak area × 100%

It is shown in Table 1 that the average ME values of corn buds, rape flowers, and marigold leaves were 84.2%, 101.8%, and 76.7%, respectively. There was a weak matrix inhibition effect in the corn buds and marigold leaves, but no obvious matrix effect in the rape flowers. This proves that the method can effectively eliminate the matrix effect and has strong versatility in the extraction of EBL from three plants.

### 2.6. Actual Determination of EBL Content in Samples

Three plant samples were studied using this method, and the content of EBL was determined via the established method. The results were repeated three times for each sample. The results showed that EBL was detected in all three plant samples, among which the contents of EBL in the maize plumule, brassica rapeseed flower, and marigold leaf were 1.253 ± 0.213, 0.637 ± 0.202, and 0.432 ± 0.071 μg/kg, respectively. After the same pretreatment, the content of EBL in the corn bud was higher than that in the rape and marigold leaves, and the content of EBL in the marigold leaves was the lowest. Maize, as an important cash crop worldwide, is of great significance in the study of plant physiology and metabolism due to its outstandingly high EBL content [30]. The results showed that the sensitivity, detection limit, and precision of this method could meet the requirements for the determination of trace EBL in different plants, and could provide a reliable analytical method for the study of EBL in maize sprouts, rape flowers, and marigold leaves.

## 3. Materials and Methods

### 3.1. Chemicals and Materials

The EBL standard (≥98%, HPLC) was purchased from Shanghai Yuanye Biotechnology Co., Ltd. (Shanghai, China). LC-grade solvents of water (H_2_O), methanol (MeOH), and acetonitrile (ACN) were purchased from Sigma-Aldrich (USA). In addition, 99% pure formic acid was purchased from Across (Livingston, NJ, USA). The SPE columns (Cleanert PEP, Cleanert ODS C18, HyperSep Retain CX, Welchrom P-SCX, CNCNW Poly-SERY PSD, CNWBOND HC-C18, Poly-Sery C18, and Poly-Sery HLB) were purchased from Tianjin Aiger Co., Ltd. (Tianjin, China). The SPE column specifications are 60 mg/3 mL.

The maize plumule was selected from the silage maize varieties mainly promoted in Gansu Province, and the seedling shoot was 18 days after the seeds germinated. The brassica rapeseed flower was from Longyou 7. The marigold leaf was from the ornamental plant in the campus of Gansu Agricultural University.

### 3.2. Sample Preparation

**Figure 6:**
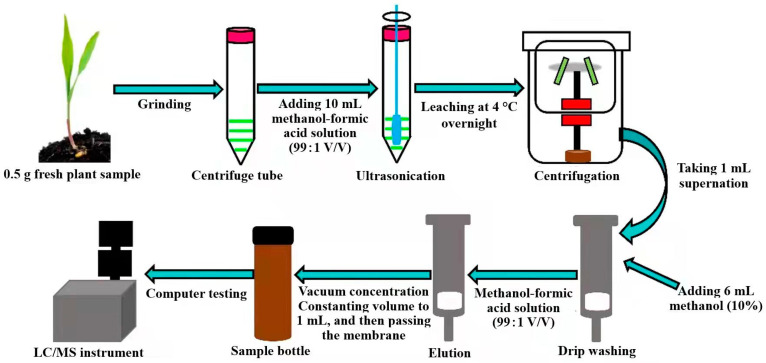
Workflow of EBL extraction in plant.

The extraction of EBL was carried out using a slightly modified version of the method described by Zhong [31] and further optimized based on this method. The specific steps were as follows: 0.5 g accurately weighed fresh plant samples (1 maize plumule, 1 rapeseed flower and 1 marigold leaf) underwent liquid nitrogen freeze-dried grinding or weighed lyophilizer freeze-dried grinding and were transferred into a 50 mL centrifuge tube. Then, 10 mL pre-cooled methanol–formic acid solution was added into the tube (99:1). The solution was ultrasonicated for 3 min at 40 Hz, and placed for 12 h in a refrigerator at 4 °C. The extraction solution was centrifuged at 14,000 r/min for 10 min, 1 mL supernatant was absorbed, and 9 mL UP H_2_O was added. (The conductivity of UP H_2_O was >18.2 MΩ/cm at 25 °C.) Purification was performed on an SPE column that had been activated in advance (with 3 mL methanol and 3 mL UP H_2_O). After adding the sample, 6 mL methanol solution of 10% was used to elute the SPE column two times. Secondly, 6 mL methanol–formic acid solution (99:1) was used for elution. The eluent was collected and concentrated to be dried with reduced pressure. Then, the methanol was reconstituted to 1 mL and filtered with a 0.22 μm organic microporous membrane for LC-MS/MS detection (Figure 6).

### 3.3. Standard Solution of EBL

Ten milligrams of compound EBL was accurately weighed using an analytical balance and separated into 10 mL volumetric flasks. It was dissolved in methanol (LC-MS grade) and the volume mas wade up to the mark to obtain a stock solution containing 1000 μg/mL. Then, 1.04 mL of aliquot from the stock solution was diluted to obtain 104 μg/mL of standard. From this solution, working standards of 104, 46.2, 13.2, 2.6, and 0.7 μg/mL were prepared via serial dilution.

### 3.4. Instrumentation

The LC–MS system consisted of a 1290 UHPLC with a binary high-pressure pump, a solvent degassing unit, and an automatic sample injector from Aigilent (Santa Clara, CA, USA). A 1290 series diode array detector (DAD) was connected in line with a bench-top mass selective detector for the 1290 series equipped with an ESI source. A series 6460C QQQ LC/MS ZORBOX Eclipse Plus C18 (2.1 mm × 50 mm, 1.8 μm) chromatographic column (Agilent, Santa Clara, CA, USA) was used. The instrument control and data processing utilities included the use of LC-MS MassHunter software. The centrifuge was a 5920R Centrifuge (German Eppendorf company), and we also used SpeedMill Plus Ultrasonic Crushers (Analyk Jena AG, Jena, Germany). The Milli-Q ultrapure water device (Millipore Corporation, USA) was used, and the conductivity of the UP H_2_O was >18.2 MΩ/cm at 25 °C. The lyophilizer was a LyoQuest-85 (Telstar Technologies, Barcelona, Spain).

### 3.5. Chromatographic Conditions

A Zorbax Eclipse Plus C18 column (50 mm × 2.1 mm × 1.8 μm, Agilent) with a solvent flow rate of 0.30 mL/min was used. The sample injection volume was set at 2.0 μL and the column temperature was set at 35 °C. The mobile phase consisted of acidified H_2_O (0.1% formic acid) as solvent A and methanol as solvent B. The solvent gradient adopted was as follows: 0–0.5 min, 10–30% B; 0.5–1 min, 30–45% B; 1–3 min, 45–90% B; 3–4 min, 90–10% B; 4–6 min, 10–10% B. Then, the final 2 min were used for column cleaning and regeneration.

### 3.6. Mass Spectrometry Conditions

MS analyses were carried out in multiple reaction monitoring (MRM) mode. The ESI-MS was operated in positive ion mode with the following instrument settings: nebulizer pressure, 0.241 MPa; gas temperature, 350 °C; gas flow, 11 L/min; capillary voltage, 4000 V; fragmentor, 100 V. Two ions for each analyte were selected, according to the specificity and sensitivity, with the primary ions used for quantification and the secondary ion providing confirmation. Other parameters are shown in Table 2.

## 4. Conclusions

In this paper, the authors extracted EBL from corn bud, rape flower, and marigold leaf, and compared the different crushing methods, various solid-phase extraction columns, the different kinds of mobile phase and mass spectrometry parameter optimization parameters during the quantitative analysis. Additionally, there is a reference scheme for extracting EBL from plants. UPLC-MS/MS has been used to solve the problem of difficult qualitative and quantitative determination of trace EBL in plant tissues. The matrix effect of EBL detection in three plants was investigated using this method, and the results showed that the matrix effect was weak or even had no effect, which demonstrated the reliability and versatility of this method. EBL detection is often quantified using the internal standard method, which is highly polar and volatile, so it is often derivatized before analysis. This method eliminates the derivation step and directly selects the characteristic ion fragment peak for qualitative and quantitative analysis, which is suitable for detection in large sample sizes and provides a theoretical basis for the comparison of agricultural samples and the study of brassinosteroids in various plants.

## Figures and Tables

**Table 1:** Recovery, linearity, and matrix effect of EBL in three plants.

Sample	Spiked Levels (μg/kg)	Recoveries (%)	RSD (%, *n* = 3)	Calibration Curves	Correlation Coefficient (R^2^)	ME (%)	LOQ (μg/kg)
Maize plumule	10	86.3	3.4	y = 19.604x + 3.657	0.9978	64.2	1.22
	50	84	1.2				
	200	96.5	0.9				
Rapeseed flower	10	88.2	4.7	y = 8.871x + 0.582	0.9997	101.8	0.36
	50	116.3	3.3				
	200	109.2	10.5				
Marigold leaf	10	85.6	4.6	y = 17.911x + 0.797	0.9996	76.7	0.95
	50	93.2	3.1				
	200	103.4	0.8				

**Table 2:** MS/MS parameters for EBL detection in plant.

Hormones	Parent Ion (*m/z*)	Fragment (V)	Product Ion (*m/z*)	Collision Energy (eV)
EBL	481.3	100	445.2 *	10
EBL	481.3	100	315.3	15

* Quantitative ion.

## Data Availability

Not applicable.

## Sample Availability

Not available.
